# Comprehension and computation in Bayesian problem solving

**DOI:** 10.3389/fpsyg.2015.00938

**Published:** 2015-07-27

**Authors:** Eric D. Johnson, Elisabet Tubau

**Affiliations:** ^1^Department of Basic Psychology, University of BarcelonaBarcelona, Spain; ^2^Research Institute for Brain, Cognition, and Behavior (IR3C)Barcelona, Spain

**Keywords:** Bayesian reasoning, mathematical problem solving, text comprehension, set-subset reasoning, numeracy, individual differences

## Abstract

Humans have long been characterized as poor probabilistic reasoners when presented with explicit numerical information. Bayesian word problems provide a well-known example of this, where even highly educated and cognitively skilled individuals fail to adhere to mathematical norms. It is widely agreed that natural frequencies can facilitate Bayesian inferences relative to normalized formats (e.g., probabilities, percentages), both by clarifying logical set-subset relations and by simplifying numerical calculations. Nevertheless, between-study performance on “transparent” Bayesian problems varies widely, and generally remains rather unimpressive. We suggest there has been an over-focus on this representational facilitator (i.e., transparent problem structures) at the expense of the specific logical and numerical processing requirements and the corresponding individual abilities and skills necessary for providing Bayesian-like output given specific verbal and numerical input. We further suggest that understanding this task-individual pair could benefit from considerations from the literature on mathematical cognition, which emphasizes text comprehension and problem solving, along with contributions of online executive working memory, metacognitive regulation, and relevant stored knowledge and skills. We conclude by offering avenues for future research aimed at identifying the stages in problem solving at which correct vs. incorrect reasoners depart, and how individual differences might influence this time point.

## Introduction

Over the past decades, there has been a growing appreciation for the probabilistic operations of human cognition. The union of highly sophisticated modeling techniques and theoretical perspectives, sometimes referred to as the “Bayesian Revolution,” is posed to bridge many traditional problems of human inductive learning and reasoning (Wolpert and Ghahramani, 2005; Chater and Oaksford, 2008; Tenenbaum et al., 2011). Despite this promising avenue, probabilistic models have acknowledged limits. One of the most prominent of these is the persistent difficulties that even highly educated adults have reasoning in a Bayesian-like manner with explicit statistical information (Kahneman and Tversky, 1972; Gigerenzer and Hoffrage, 1995; Barbey and Sloman, 2007), including individuals with advanced education (Casscells et al., 1978; Cosmides and Tooby, 1996), higher cognitive capacity (Lesage et al., 2013; Sirota et al., 2014a), and higher numeracy skills (e.g., Chapman and Liu, 2009; Hill and Brase, 2012; Johnson and Tubau, 2013; Ayal and Beyth-Marom, 2014; McNair and Feeney, 2015). Rather than contradicting Bayesian models of reasoning, however, less than optimal inferences over explicit verbal and numerical information result in large part from the relatively recent cultural developments of these symbolic systems, far too little time for evolution to have automated this explicit reasoning capacity.

In the present review, we focus on Bayesian word problems, or the *textbook-problem paradigm* (Bar-Hillel, 1983), where a binary hypothesis and observation (e.g., the presence of a disease, the results of a test) are verbally categorized and numerically quantified within a hypothetical scenario. We use the term “Bayesian word problems” to refer to tasks in which these explicitly summarized statistics are provided as potential input for a Bayesian inference in order to derive a posterior probability (these correspond to “statistical inference” tasks in Mandel, 2014a). Specifically, the base rate information (e.g., the probability of having a disease) has to be integrated with the likelihood of a certain observation (e.g., the validity of a diagnostic test, reflected in a hit rate, and false-positive rate) to arrive at precisely quantified Bayesian response (e.g., the probability of having the disease conditioned on a positive test). Hence, these problems reflect situations of *focusing* (rather than *updating per se*), where an initial state of knowledge is refined, or re-focused, in an otherwise stable universe of possibilities (Dubois and Prade, 1992, 1997; Baratgin and Politzer, 2006, 2010). Given this static coherence criterion of these word problems, the normative view of additive probability theory holds (Kolmogorov, 1950), and so Bayes' rule is the most appropriate normative standard for assessing performance (see Baratgin, 2002; Baratgin and Politzer, 2006, 2010)^1^.

Some have argued that Bayesian word problems may in fact have little to do with “Bayesian reasoning” in the sense that they do not necessarily require updating a previous belief (see Koehler, 1996; Evans et al., 2000; Girotto and Gonzalez, 2001, 2007; Mandel, 2014a; Girotto and Pighin, 2015). This sentiment reflects a gradual shift from using these tasks to understand how well (or poorly) humans update the probability of a hypothesis in light of new evidence, or how experienced physicians diagnose disease given a specific indicator (Casscells et al., 1978; Eddy, 1982), to the task features and individual differences associated with reasoning outcomes, which are often found to depart from the Bayesian ideal (Barbey and Sloman, 2007; Navarrete and Santamaría, 2011; Mandel, 2014a). We take this descriptive-normative gap to be our general question: *Why do people tend to deviate, often systematically, from the normative standard prescribed by Bayes' rule*?

Fortunately not all is lost, and a variety of factors are increasingly understood which can be manipulated to facilitate Bayesian responses from floor to near ceiling performance. In what follows, we first aim to clarify some frequently confused terms, isolate key factors influencing performance, and point out some limitations of typically contrasted theoretical views. We then highlight some mutually informative parallels between research and theory on Bayesian inference tasks, and the literature on mathematical problem solving and education. Finally, we discuss how these separate, but complimentary, views on reasoning and mathematical cognition can provide some general processing considerations and new methodologies relevant for understanding why human performance falls short of Bayesian ideals, and how this gap might be reduced.

## Natural frequencies: from base-rate neglect to nested-sets respect

In the present section we explore the Bayesian reasoning task, using a variant of the classic medical diagnosis problem (Casscells et al., 1978; Eddy, 1982) as a general point of reference. We center on the natural frequency effect—a facilitator of both representation *and* computation—and the debate which has surrounded it for nearly two decades. We highlight the general consensus on the benefits of making nested-set structures transparent, before turning to other processing requirements needed for transforming presented words and numbers into a posterior Bayesian response in the following section.

### Poor reasoning and base-rate neglect

“In his evaluation of evidence, man is apparently not a conservative Bayesian: he is not Bayesian at all.”

Kahneman and Tversky (1972 p. 450)

Although Bayesian norms have been around since the 18th century (Bayes, 1764), it was not until 200 years later that psychological research adopted these standard as the benchmark against which to measure human reasoning ability. As exemplified in the quote above, early results were not too promising. In the heyday of the heuristics-and-biases paradigm, one of medicine's most coveted journals, the *New England Journal of Medicine*, published a study where a group of medically trained physicians were given the following problem (Casscells et al., 1978):

If a test to detect a disease whose prevalence is 1/1,000 [**BR**] has a false positive rate of 5% [**FPR**], what is the chance that a person found to have a positive result actually has the disease, assuming that you know nothing about the person's symptoms or signs?___%^2^.

This medical diagnosis problem asks for the probability (chance) that a person actually has the disease (the hypothesis) given a positive test result (the data), a task of which physicians should be reasonable adept. The results, however, were not very encouraging, with only 18% of the physicians answering with the Bayesian response of 2%. Forty-five percent of them, on the other hand, answered “95%,” which appeared to completely ignore the base rate presented in the problem—the fact that only 1 in 1000 people actually have the disease. Similar results were reported a few years later by Eddy (1982). Evidence was accordingly interpreted to show that humans tend to neglect crucial information (such as base rates), while instead focusing on the similarity of target data to prototypical members of a parent category (for reviews see Kahneman et al., 1982; Koehler, 1996). This was part of a larger explanatory framework which emphasized limited cognitive processing capacity, where mental shortcuts, or heuristics, are employed to alleviate the burden of cognitively demanding tasks, including those that may be more optimally answered with formal calculations (e.g., Kahneman, 2003). However, “base-rate neglect” as a general explanation has been critiqued on theoretical and methodological grounds (Koehler, 1996), which is further supported by the observation that typical errors in Bayesian word problems tend to be a function of the question format, with base-rate-only responses often reported (e.g., Gigerenzer and Hoffrage, 1995; Mellers and McGraw, 1999; Evans et al., 2000; Girotto and Gonzalez, 2001).

### The natural frequency effect: Evolution and computation

At a time when pessimism dominated the landscape of the cognitive psychology of reasoning, Gigerenzer and Hoffrage (1995) and Cosmides and Tooby (1996) offered hope for the human as statistician, along with a strong theoretical agenda (see also Brase et al., 1998). Consider this *frequency* alternative to the Casscells et al. (1978) medical diagnosis problem presented above:

1 out of every 1000 [**BR**] Americans has disease X. A test has been developed to detect when a person has disease X. Every time the test is given to a person who has the disease, the test comes out positive [**HR** = **1**]. But sometimes the test also comes out positive when it is given to a person who is completely healthy. Specifically, out of every 1000 people who are perfectly healthy, 50 of them test positive [**FPR**] for the disease.

Imagine that we have assembled a random sample of 1000 Americans. They were selected by a lottery. Those who conducted the lottery had no information about the health status of any of these people. Given the information above, on average, how many people who test positive for the disease will actually have the disease? __ out of __.

**Figure 1:**
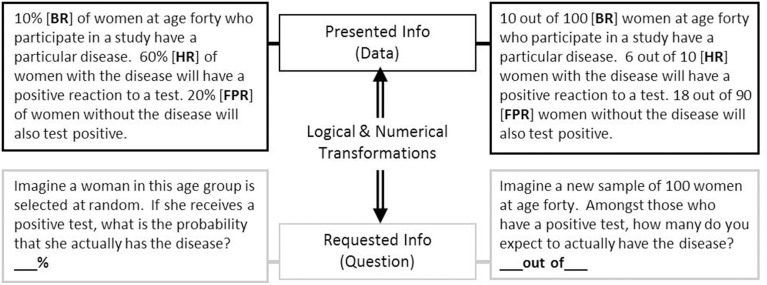
**Examples of the medical diagnosis problem, presented with ***normalized*** numerical information (left) and with ***natural frequencies*** (right)**. If not otherwise indicated, other tables, figures, and examples in the text refer to the numerical information in this figure.

Performance on this problem was found to elicit a correct response rate of 72% by Cosmides and Tooby (1996, study 2), remarkably higher than the 18% reported by Casscells et al. with the formally analogous information shown above. In a similar vein, Gigerenzer and Hoffrage (1995, 1999) reported success rates near 50% across a variety of problems presenting *natural frequencies*, compared to 16% with their probability versions. Examples of similar problems presenting natural frequencies and normalized data are shown in Figure 1.

**Figure 2:**
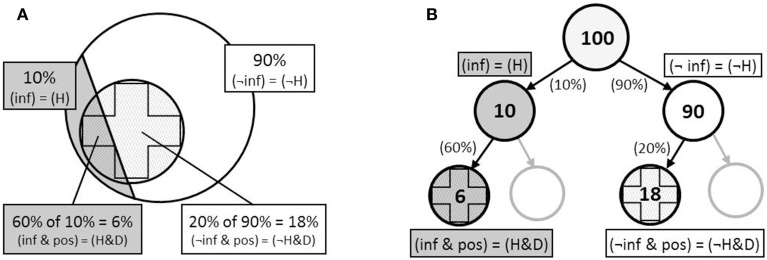
**Representations of the Bayesian tasks presented in Figure 1 as (A) integrated nested sets, and (B) frequency tree, where (***H***) the hypothesis = infected (inf); and (***D***), the data = positive test (pos)**.

The initial explanations offered for these effects can be divided in two strands: *Evolution* and *computation*. According to Cosmides and Tooby, evolution endowed the human mind with a specialized, automatically-operating frequency module for making inferences over countable sets of objects and events, but which is ineffective for computing single-event probabilities. By tapping into this module naïve reasoners can solve frequency problems, while they fail on probability problems because this module cannot be utilized. Relatedly, Gigerenzer and Hoffrage suggested that reasoning performance depended on the mesh between the presented problem data (the structure of the task environment) and phylogenetically endowed cognitive algorithms for *naturally sampled* information (Kleiter, 1994; Figure 2B), which leads to a similar suggestion that explicit numerical reasoning would utilize the same cognitive processes used for reasoning based on information experienced over time, provided the external input matched the internal algorithm. Unlike Cosmides and Tooby (1996) and Gigerenzer and Hoffrage (1995) did not specifically argue that the mind is unable to deal with probabilities of single events, and in fact their computational account predicted quite the opposite (see their study 2 and “prediction 4”).

The more pertinent claim of Gigerenzer and Hoffrage (1995), however, was their *computational* analysis, which focused on the difference between the information provided in the problem and its proximity to the Bayesian solution. With normalized information (e.g., percentages; see Table 1), the following computation is necessary to arrive at a Bayesian response, where *H* is the hypothesis (having the disease) and *D* is the data (testing positive):

$$\begin{array}{l} p(H|D)=\frac{p\left(H\&D\right)}{p\left(D\right)}=\frac{p(H)p(D|H)}{p(H)p(D|H)+p(\neg H)p(D|\neg H)}\\ \;=\frac{\left(0.1)(0.6\right)}{\left(0.1)(0.6)+(0.9)(0.2\right)}=“25\%” \end{array}$$

**Table 1:** **Key dimensions along which a Bayesian word problem may vary**.

**Dimension**	**Description and variables**
Numerical format	The format of the presented numerical information: *Whole number integer pairs* (e.g., 10 of 100) vs. *Normalized* (e.g., 10%, 0.1). Formats can be mixed within a single problem.
Question format	The format of the requested response, typically: *Integer pair* (e.g., “__ out of __”) vs. *Normalized* (e.g., “__%”).
Number of events	*Single-event* (e.g., probability, chance) vs. *Set of events* (e.g., individuals, chances). Can apply to both the presented data (“information type”) or to the information requested (“task domain”). Often confused with numerical format, but these are orthogonal issues.
Sampling structure	The particular categorical-numerical information used to express the hit rate and false-positive rate, typically: *Natural* (*H*&*D*, ¬*H*&*D*; also partitive, transparent, conjunctive, joint) vs. *Normalized* (*D|H* and *D*|¬*H*; also non-partitive, relative frequencies, conditional).
Natural frequencies	A problem format which presents *whole numbers* in a *natural sampling* structure (e.g., *H*&*D*, ¬*H*&*D*), and requests responses as an *integer pair*.
Normalized problems	A problem which presents normalized numerical formats (percentages, decimals), a normalized sampling structure (i.e., with conditional or non-conjunctive information), and/or which requests information in a normalized format (a ratio as a single value, not integer pair).
Context	Scenario of the problem. For example, medical (infection, test); cab (accident, color).
Irrelevant info	Descriptive information that is not relevant for solving the task. Numbers that are not needed for computing the normative response.
Mental steps	The number of steps required to compute the response, given the specific numbers presented in the problem. For example, in Figure 1, the number “24” (total positive tests, *D*) is needed but not presented, and must be calculated from (6 + 18) = 1 numerical step.
Compatibility	Correspondence between the presented and requested data, including numerical and question formats, also sample sizes.

With natural frequencies, on the other hand, all numerical information is absolutely quantified to a single reference class (namely, the superordinate set of the problem; “100 people” in Figure 1; see also Figure 2B), where categories are naturally classified into the joint occurrences found in a bivariate 2 × 2 table [e.g., (*H*&*D*), (¬*H*&*D*)]. In this case, the conditional distribution does not depend on the between-group (infected, not infected) base rates, but only on the within-group frequencies (hit rate, false-positive rate). Accordingly, base rates can be ignored, numbers are on the same scale and can be directly compared and (additively) integrated, and the required computations are reduced to a simpler form of Bayes rule:

$$\begin{array}{l} p(H|D)=\frac{p\left(H\&D\right)}{p\left(D\right)}=\frac{p\left(H\&D\right)}{p\left(H\&D\right)+p(\neg H\&D)}\\ \;=\frac{6}{6+18}=“6\;out\;of\;24” \end{array}$$

### Thinking in sets: Comprehension and manipulation of nested-set structures

While the computational simplification afforded by natural frequencies was clear, critiques of the evolutionary view came quickly. Over the ensuing decade, a number of studies appeared which argued that the “frequency advantage” was better described as a “structural advantage” (Macchi, 1995; Macchi and Mosconi, 1998; Johnson-Laird et al., 1999; Lewis and Keren, 1999; Mellers and McGraw, 1999; Evans et al., 2000; Girotto and Gonzalez, 2001, 2002; Sloman et al., 2003; Yamagishi, 2003; Fox and Levav, 2004). More specifically, these studies suggested that the benefit of natural frequencies was not in the numerical format *per se* (frequencies vs. percentages), nor the number of events being reasoned about (sets of individuals vs. single-event probabilities), but rather in the clarification of the abstract nested-set relationships inherent in the problem data, which helps reasoners to form appropriate models of relevant information. The nested-set structure of these problems is illustrated in Figure 2, where it can be seen that the relations between categories—people *infected* (*H*) and *not infected* (¬*H*) testing *positive* (*D*) for a disease—can be represented spatially as a hierarchical series of nested sets.

Clearly, the quantitative relationships amongst subsets are more transparently afforded with natural frequencies compared to normalized percentages. This general view emphasizing *representational* facilitation has come to be known as the *nested-sets hypothesis*, originally proposed by Tversky and Kahneman (1983), and which has since been variously expressed by a number of authors. For example, Mellers and McGraw (1999) concluded that natural frequencies are “advantageous because they help people visualize nested sets, or subsets relative to larger sets” (p. 419). Girotto and Gonzalez (2001) attributed successful reasoning to problem presentations which “activate intuitive principles based on subset relations” (p. 247). For Evans (2008), “what facilitates Bayesian reasoning is a problem structure which cues explicit mental models of nested-set relations” (p. 267). And as stated by Barbey and Sloman (2007, p. 252): “the mind embodies a domain general capacity to perform elementary set operations and that these operations can be induced by cues to the set structure of the problem.” Although these suggestions are not without limitations (discussed below), proponents of the nested-sets hypothesis helped identify a key strategy that reasoners (naïve to Bayes rule) *can* use to arrive at a Bayesian response: *Thinking in sets*. That is, in the absence of formal knowledge of how to optimally combine conditional probabilities, reasoners can still solve these tasks by considering the problem as overlapping sets of data, namely, as a focal subset of infected people out of the reference set of people who test positive: (*H*&*D*)/(*D*).

Contemporary discussions explaining Bayesian facilitations continue to be framed in terms of a nested-sets (or domain general) contra an ecological rationality (or frequency/format-specific) debate (e.g., Navarrete and Santamaría, 2011; Hill and Brase, 2012; Lesage et al., 2013; Brase, 2014; Sirota et al., 2014a,b, 2015a,b; Brase and Hill, 2015). We assume that theorists on both sides of the divide are more interested in finding out how to improve Bayesian reasoning and why these facilitations work, rather than simply promoting a preferred position. We also believe that, in general, these perspectives may in some regards be more complimentary than adversarial. Accordingly, we think that it is important to acknowledge what these views have in common, where the relevant differences between these views lie, and whether either view can fully account for empirical data.

To begin, it should by now be well understood that *natural frequencies* do not simply refer to the use of frequency formats, but essentially refer to problem structure as well (Gigerenzer and Hoffrage, 1999, 2007; Hoffrage et al., 2002; for concurrence see Barbey and Sloman, 2007, response R3). Both the natural frequency and nested-sets views agree that frequencies that do not conform to a natural sampling, or partitive, structure are not much better than percentage formats (Evans et al., 2000; Girotto and Gonzalez, 2001; Sloman et al., 2003). Where these two views primarily diverge is in how comfortable they are making precise predictions based on evolutionary claims. For the moment we suggest putting the evolutionary claims aside, and instead focusing on two points of commonality. First, natural frequencies (or problems presenting a “partitive” or “nested-set” structure, or conforming to the “subset principle”; see Brase and Hill, 2015) are widely agreed to be the most general and robust facilitator of Bayesian-like performance. Second, natural frequencies facilitate both representation *and* computation.

We suggest that in order to advance the discussion, we need to move away from the standard “natural frequency vs. nested-sets” debate and instead consider the processing requirements, and corresponding difficulties, given a particular problem presentation (see also McNair, 2015; Vallée-Tourangeau et al., 2015b). In the following section we note key performance variables and often confused issues, and review available evidence looking separately at problems presenting and requesting normalized vs. natural frequency information.

## The Bayesian problem: from words and numbers to meaningful structures

Table 1 presents some commonly used terms that are often used in different ways and which frequently lead to confusion. Below we briefly highlight the most frequently confused factors (see also Barton et al., 2007).

First, numerical format and the number of events are fully orthogonal dimensions. Normalized formats (e.g., percentages, decimals) can express single-event probabilities (e.g., “10% chance of infection”) or proportions of a set (e.g., “10% of people are infected”), and whole numbers can be used to express frequencies (e.g., 10 of 100 people) or single events (10 of 100 chances). This applies both to the information presented in the text and requested in the question.

Second, the “sampling structure” (also referred to as “information structure” or “menu”) refers to the specific categorical-numerical information used to express the hit rate and false-positive rate, and is also orthogonal to the above two distinctions (numerical format, number of events). Typically, this refers to the presentation of the conjunctive/joint events [(*H*&*D*) and (¬*H*&*D*)] vs. the conditional/normalized data [(*D*|*H*) and (*D*|¬*H*), along with the base rates (*H*) and (¬*H*)]. Any of these categories can be quantified with either frequencies or normalized formats.

Finally, throughout this review we use the term “natural frequencies” to refer to problems which (1) present *whole numbers* (2) in a *natural sampling* (or *partitive*) structure (specifically, one which directly presents *H*&*D* and ¬*H*&*D*), and (3) request *responses as an integer pair*. We acknowledge that on some accounts natural frequencies may refer only to the initial problem data independent of the question format. However, as we review, the primary benefits of natural frequencies hold only when the question also requests a pair of integers (Ayal and Beyth-Marom, 2014), and therefore for ease of exposition we use *natural frequencies* only when all three conditions are present (unless otherwise stated). In contrast, we refer to “normalized” problems as those which do not meet these three criteria (see Table 1).

Why do natural frequencies facilitate Bayesian-like responses? In order to answer this question, we have to understand what was so hard in the first place. That is, a facilitation must always be made relative to some initial point, and it is therefore important first to understand why normalized versions are so difficult. We will then be in a better position to understand the facilitating effects of natural frequencies, and more generally why even clearly presented problems can still be so difficult for many reasoners. In the remainder of this section we therefore review factors that have been shown to facilitate, or impair, Bayesian-like reasoning with problems presenting normalized information or natural frequencies separately.

### Reasoning with normalized formats

Reasoning with normalized formats is notoriously difficult. However, observing that more “transparent” problems facilitate performance does not necessarily imply that normalized versions are hard simply because the presented data is more difficult to represent. As reviewed below, the difficulty of these problems cannot be reduced to a single (representational or computational) factor. Although some improvements have been observed with visual diagrams and verbal manipulations to the text and question, as well as for individuals with higher cognitive and numerical ability, all of these are limited in their effectiveness.

#### Visual representations

Some evidence suggests that visual aids may boost performance with normalized data, which presumably help reasoners to appreciate nested-set relations (for recent reviews see Garcia-Retamero and Cokely, 2013; Garcia-Retamero and Hoffrage, 2013). For example, Sedlmeier and Gigerenzer (2001) showed that training individuals to use frequency trees could have substantial and lasting effects on complex Bayesian reasoning scenarios. Mandel (2015) more recently showed that similar instructions on information structuring improve the accuracy and coherence of probability judgments of intelligence analysts. Recent work by Garcia-Retamero and Hoffrage (2013) also showed substantial benefits of visual aids with probability information. Yamagishi (2003) found that both a roulette-wheel diagram and a frequency tree led to large improvements with information presented as simple fractions (e.g., 1/4, 1/3, 1/2) in the gemstone problem. Sloman et al. (2003) also showed that a Euler circle diagram marginally facilitated performance on a probability version of the medical diagnosis problem. However, in a counterintuitive Bayesian task, the Monty Hall dilemma, Tubau (2008) found no facilitation of a diagrammatic representation of the problem. Overall, while visual diagrams may help with normalized data under some conditions, this facilitation is typically very modest, although instruction or training in information re-representation may be an effective way to improve reasoning in some populations.

#### Verbal formulation and irrelevant information

There is evidence that reasoning with normalized data can be improved by manipulating the verbal structure of the problem, independent of the numbers provided (Macchi, 1995; Sloman et al., 2003; Krynski and Tenenbaum, 2007; Hattori and Nishida, 2009; Johnson and Tubau, 2013; Sirota et al., 2014a). For example, Macchi (1995) showed how questions which were slightly reformulated to focus on individuating (vs. base-rate) information increased (or reduced) the number of base-rate neglect responses. Sloman et al. (2003, exp. 1) found differences between three numerically identical versions of the medical diagnosis problem, but which varied in the particular wording (or “transparency of nested-set relations”) used to transmit the problem data (see also Sirota et al., 2014a, exp. 2). They additionally reported that irrelevant numbers impaired performance, but only with normalized versions (exp. 4B). Johnson and Tubau (2013) also found that simplifying the verbal complexity improved Bayesian outcomes with probabilities, but this was restricted to higher numerate reasoners^3^.

Krynski and Tenenbaum (2007) also showed that manipulating verbal content, independent of the numbers, can boost performance. They suggested that reasoners supplement the statistical data presented in the problem with prior world knowledge (of causal relations), and therefore Bayesian reasoning could be enhanced by presenting false-positive rates in terms of alternative causes. Simply providing a cause for the false-positive rate (e.g., “the presence of a benign cyst” in the medical diagnosis context) boosted performance from approximately 25 to 45%, some of the highest performance reported with normalized data in the absence of visual cues. It should be noted, however, that McNair and Feeney (2015; see also 2014) were unable to fully replicate this effect, though they did find evidence that higher numerate reasoners significantly benefitted from a clearer causal structure with normalized information. The participants in Krynski and Tenenbaum's study consisted of undergraduate and graduate students at MIT, who are presumably a more mathematically sophisticated group, which may help to account for the consistent main effect of causal structure in their studies (cf. Brase et al., 2006). This suggests that providing “alternative causes” helped draw attention to the often neglected false-positive data (Evans et al., 2000), which could then be taken advantage of by individuals possessing the requisite numerical skills.

#### Computation

Normalized versions typically require multiple steps using fraction arithmetic. Despite claims in the reasoning literature that the fraction arithmetic (multiplying and dividing percentages) required in these tasks in relatively easy (e.g., Johnson-Laird et al., 1999; Sloman et al., 2003), there is indeed substantial evidence that many people lack the requisite conceptual and/or procedural knowledge to correctly carry out these computations (Paulos, 1988; Schoenfeld, 1992; Mayer, 1998; Ni and Zhou, 2005; Reyna and Brainerd, 2007, 2008; Siegler et al., 2011, 2013). Indirect evidence for the difficulty performing multiplicative integrations on normalized problem data is also suggested in Juslin et al. (2011), where it was proposed that reasoners default to less demanding linear additive integrations in the absence of requisite knowledge, cognitive resources, or motivation (see also Juslin, 2015).

An informative result was provided in Ayal and Beyth-Marom (2014). Participants were provided probability information in a percentage format, but the problems were manipulated so that *p*(*H*|*D*) could be computed via a single whole-number subtraction (1 − *p*(¬*H*|*D*) = 100 − 92% = 8%). Responses were requested either as a percentage (“compatible”) or as frequencies (“incompatible”). In the compatible condition, nearly 80% of higher numerate and around 60% of lower numerate reasoners correctly computed *p*(*H*|*D*); in the incompatible condition, around 70% or of higher numerate and around 34% of lower numerate individuals responded correctly. On the one hand, this expectedly demonstrates that higher numerate individuals are more able to translate between numerical formats. More importantly, however, the high proportion of correct responses, even by reasoners with lower numeracy, demonstrates that the participants in these studies are not inherently unable to understand set relations presented as standardized probabilities. It also shows that the typical computational demands (steps and/or type) with normalized formats may in fact impede Bayesian-like responding (cf. Juslin et al., 2011). It should also be noted, however, that this condition does not require reasoners to understand embedded sets of information (i.e., to simultaneously consider and integrate base rates and diagnostic information); rather, they are simply required to represent the complement of a whole. This implies that the representational difficulty on standard Bayesian problems is not specific to the structure of the data itself, but rather to the relation between the presented and requested information (see also Section Common Processing Demands: Quantitative Backward Reasoning).

#### Number of events

Existing research suggests that presenting or requesting single-event probabilities vs. a proportion of a sample (or relative frequencies) with percentages may have little impact on Bayesian responding with normalized data, all else held constant. For example, Gigerenzer and Hoffrage (1995, study 2) found no differences when presenting the data as either relative frequencies with percentages (as in Figure 1) vs. single-event probabilities when the question requested a probability. Likewise, Evans et al. (2000, study 2) found no differences with questions requesting a single-event probability vs. a proportion of a sample from data presented as relative frequencies with percentages. While this may be taken as evidence that Bayesian reasoning with percentage information is independent of the number of events referred to, this does not necessarily imply that single-event probabilities are as easily understood as relative frequencies expressed as percentages (e.g., Brase, 2008, 2014; Sirota et al., 2015a; see discussion of “*Chances*” below in Section Reasoning with Natural Frequencies). Recent re-analyses of data from Gigerenzer and Hoffrage (1995) show that problems focusing on individuals (compared to samples, or “numbers”) indeed lead to fewer Bayesian responses (Hafenbrädl and Hoffrage, 2015).

#### Individual differences

The general finding from individual differences research is that higher cognitive ability, disposition toward analytical thinking, and numeracy level can lead to improved reasoning under some conditions, but to a limited extent (Table 2). Sirota et al. (2014a) found that general intelligence (Raven et al., 1977), as well as preference for rational thinking (REI; Pacini and Epstein, 1999), uniquely predicted performance with single-event probabilities. Results of McNair and Feeney (2015) also suggested a significant association between Raven's matrices and performance on normalized Bayesian versions, but an absence of association between the latter and REI. Of note, two studies have reported a lack of association between normalized Bayesian problems and the cognitive reflection test (CRT; Frederick, 2005), a measure of the tendency to suppress initial intuitions and engage in more demanding analytical processing (Lesage et al., 2013; Sirota et al., 2014a). Together, these results suggest that providing the posterior Bayesian ratio with normalized information will necessarily depend on high levels of cognitive ability *and* numeracy. Without these basic requisites, reflective thinking or disposition toward analytical thinking are likely to be of little help (De Neys and Bonnefon, 2013). It is also important to note that even the performance of “higher” ability individuals typically remains quite low. Nevertheless, few studies have directly investigated these factors and results have been mixed, therefore more research is needed to clarify when (and in what combination) individual differences measures are likely to be relevant (proposals of the relative dependencies of these factors can be found in Stanovich, 2009; Klaczynski, 2014; see also Thompson, 2009).

**Table 2:** **Summary of significant individual differences effects reported in Bayesian word problems presenting normalized information or natural frequencies**.

	**Numeracy/education**	**IQ-raven**	**CRT ^I^**	**Thinking disposition**
NORMALIZED VERSIONS^*^				
Chapman and Liu, 2009	No			
Siegrist and Keller, 2011	Yes/No ^a^			
Hill and Brase, 2012	No			
Garcia-Retamero and Hoffrage, 2013	Yes			
Johnson and Tubau, 2013	Yes/No ^a^			
Lesage et al., 2013			No	
Sirota et al., 2014a		Yes	No	Yes/No ^b^
Ayal and Beyth-Marom, 2014	Yes ^c^			
McNair and Feeney, 2015	Yes/No ^d^	Yes		No ^e^
**NATURAL FREQUENCIES**				
Brase et al., 2006	Yes			
Chapman and Liu, 2009	Yes			
Sirota and Juanchich, 2011	Yes		Yes	
Siegrist and Keller, 2011	Yes/No ^f^			
Hill and Brase, 2012	Yes			
Garcia-Retamero and Hoffrage, 2013	Yes			
Johnson and Tubau, 2013	Yes/No ^g^			
Lesage et al., 2013			Yes	
Sirota et al., 2014a		Yes	Yes	Yes/No ^b^

*Note that variation exists between the specific context and numbers used across studies, as well as specific measures and criteria used to determine low vs. high performers (see text for additional details, and original articles for full problems and explanations)*.

* *It is important to note that YES with normalized versions does not imply “good” reasoning, with most higher ability participants typically below 30% correct response*.

I *CRT, Cognitive Reflection Test (Frederick, 2005)*.

a *YES with simple versions; NO with complex versions (floor effect)*.

b *YES with REI (rational-experiential inventory; rational thinking); NO with CAOMTS (actively open-minded thinking)*.

c *Information was normalized, but problems manipulated to require only simple single-step arithmetic*.

d *Higher numerate benefited more from causal manipulation used in Krynski and Tenenbaum (2007)*.

e *NO with REI*.

f *YES in study 1; NO in study 2 (though clear trend)*.

g *YES with complex text; NO with short, simple text*.

### Reasoning with natural frequencies

Providing information as natural frequencies (or naturally partitioned sets of chances) is widely hailed as the most effective and robust facilitator of Bayesian-like reasoning. Nevertheless, between-study performance varies widely, and success even with natural frequencies generally remains rather unimpressive (see Newell and Hayes, 2007; Girotto and Pighin, 2015; McNair, 2015). Why do so many individuals still fail to solve these problems even when the structures of these tasks are made “transparent?”

#### Computation

In their standard form, natural frequencies typically require only a single addition of two whole numbers to construct the needed reference set (*D*), and the selection of the joint occurrence (*H*&*D*) directly provided in the text, to answer the Bayesian question “*(H*&*D) out of (D)*.” Clearly, the whole-number arithmetical demands of the task are manageable by the undergraduate students tested in most studies, as well as by children (Zhu and Gigerenzer, 2006). At the same time, there is also evidence that many people either lack the cognitive clarity or are unwilling to invest the needed cognitive effort into even the simplest whole number arithmetic (addition, subtraction). For example, confirming their “mental steps hypothesis,” Ayal and Beyth-Marom (2014) showed that performance drops sharply when more than a single numerical operation is required, even if these operations are little more than a series of simple additions. Related findings were observed in the “defective nested sets” study reported in Girotto and Gonzalez (2001, study 5) which presented a partitive structure [but with (¬*H*&¬*D*) instead of (¬*H*&*D*)], but which required an additional subtraction to solve. Together, these findings demonstrate that natural frequency facilitations are not simply about the clarity of the presented data, but are also about how easily the specifically presented components allow reasoners to generate the Bayesian solution (see also Barbey and Sloman, 2007).

#### Verbal formulation and irrelevant information

As with normalized versions, manipulating the verbal context of a problem to align with existing world knowledge can improve performance. For example, Siegrist and Keller (2011, study 4; see also Sirota et al., 2014a, study 2; Chapman and Liu, 2009) showed that a less educated group from the general population was more than twice (13 vs. 26%) as likely to solve a “social” problem (people lie, have red nose) vs. a “medical” problem (have cancer, test positive). They suggested this group may focus on specific task information in a real-world context, and might have assumed they did not know enough about cancer or medical tests to solve the problem. There is also evidence that performance, especially by lower numerate reasoners, is impaired by the presence of unnecessarily descriptive words in the text (Johnson and Tubau, 2013). Other verbal manipulations, such as clarifying the meaning of “false positive,” have also been suggested to improve performance (Cosmides and Tooby, 1996; Sloman et al., 2003; see also Fox and Levav, 2004). Sloman et al. (2003) found that irrelevant numbers in the problem did not impair performance with transparent frequency problems, and suggested that a frequency format “makes it easier for people to distinguish relevant from irrelevant ratios” (p. 304). However, a very frequently reported error with natural frequencies is that reasoners use the superordinate value of the problem or the new reference class presented in the question as the denominator in their response (e.g., “100” in Figure 1; see Gigerenzer and Hoffrage, 1995; Macchi and Mosconi, 1998; Mellers and McGraw, 1999; Evans et al., 2000; Girotto and Gonzalez, 2001; Brase et al., 2006; Zhu and Gigerenzer, 2006), suggesting that irrelevant numbers may indeed bias responses on simple natural frequency problems.

#### Visual representations

Sloman et al. (2003) also found that Euler circles did not further enhance performance with their frequency problem, and suggested that visuals only facilitate if nested-set relations are not already clear (see also Cosmides and Tooby, 1996). In contrast, Yamagishi (2003) found improvements on a natural frequency gemstone problem with a roulette-wheel diagram. Brase (2009) did not find a benefit of a Venn diagram with chance versions in a natural frequency structure, however an icon display did provide an additional benefit beyond the frequency format (see also Brase, 2014). Complementary results by Garcia-Retamero and Hoffrage (2013) also showed benefits of visual aids above and beyond the use of natural frequencies. Garcia-Retamero et al. (2015) further showed that visual aids are particularly beneficial to lower numerate reasoners, and may also improve their metacognitive judgment calibration. Contrasting with the above, Sirota et al. (2014b) failed to find a benefit with several types of visuals. In brief, while some facilitation with visual aids has been reported with natural frequencies, current evidence is conflicting and suggests that other factors are likely interacting with the effectiveness of these aids.

#### Chances

Although initial reports implied that naturally sampled chances were as easily represented as naturally sampled frequencies (Girotto and Gonzalez, 2001), more recent studies show that this might not be the case (Brase, 2008, 2014; Sirota et al., 2015a). This would be in line with more general literature on the difficulties that people have learning and understanding probabilities (e.g., Garfield and Ahlgren, 1988; Gigerenzer et al., 2005; Morsanyi et al., 2009; Morsanyi and Szücs, 2015). It would also imply that the lack of difference between probability and proportion formulations with normalized data (see “*Number of Events*” above in Section Reasoning with Normalized Formats) is not because these formats are equally well (or poorly) understood, but rather that the difference is being masked by another more fundamental difficulty with normalized information (carrying out fraction arithmetic; understanding or identifying the requested relations). Interestingly, participants who interpret naturally sampled “chances” as frequencies outperform those individuals who interpret them as single-event probabilities (Brase, 2008, 2014). Also of interest, more recent evidence suggests the relevant “interpretation” may be at the problem level (in terms of set relations) rather than at the format level (in terms of frequencies) (Sirota et al., 2015a).

#### Individual differences

It has been argued that the wide variability reported with natural frequency problems can be attributed to individual differences in ability or motivation (Brase et al., 2006; Barbey and Sloman, 2007). In line with this suggestion (and summarized in Table 2), better performance with natural frequencies has been observed by individuals higher in *cognitive reflection* (Sirota and Juanchich, 2011; Lesage et al., 2013; Sirota et al., 2014a; measured with the CRT); *fluid intelligence* (Sirota et al., 2014a; measured with Raven's matrices); preference for *rational thinking* (Sirota et al., 2014a; measured with the REI), *education level* (Brase et al., 2006; Siegrist and Keller, 2011; though see Hoffrage et al., 2015), and *numeracy* (Chapman and Liu, 2009; Sirota and Juanchich, 2011; Hill and Brase, 2012; Garcia-Retamero and Hoffrage, 2013; Johnson and Tubau, 2013; Garcia-Retamero et al., 2015; McNair and Feeney, 2015). These higher ability individuals often perform quite well, although the success of even these more capable individuals varies widely across studies.

While some of the between-study variation with natural frequencies can be captured by these individual differences factors, the strong relations observed with “higher ability” reasoners also raises some questions. Why are general intelligence, cognitive reflection, and numeracy so consistently relevant on such an arithmetically simple task, especially one in which the “structural transparency” of the task is such a well-toted facilitator? Indeed, due to the base-rate preservation, there is no need for a fully fleshed out representation of the entire problem structure, and attention need only be allocated to two pieces of information, (*H*&*D*) and (¬*H*&*D*). Together, performance on these “simple” problems implies that, beyond simple text processing and whole-number arithmetic, there may be a particular logical difficulty inherent in these problems that is often overlooked.

### Common processing demands: quantitative backward reasoning

Early studies of Bayesian inference with the medical diagnosis task were specifically directed at understanding how individuals (e.g., physicians) diagnosis disease given a prior distribution and an imperfect predictor (a test result) (Casscells et al., 1978; Eddy, 1982). More recently, logical and set operations have been identified as a useful strategy for performing these Bayesian inferences (e.g., Sloman et al., 2003), however, we believe that the particular nature of the required set operations has been underemphasized in recent studies. More specifically, we suggest that a particularly difficult stage of Bayesian problem solving is performing a *backward* (diagnostic) inference (van den Broek, 1990; Oberauer and Wilhelm, 2000; Lagnado et al., 2005; Fernbach et al., 2011; Sloman and Lagnado, 2015)—in the medical diagnosis problem, working backward from a positive test result (effect) to the likelihood of being infected (cause), when information is provided in the forward cause → effect direction. For example, querying the model in Figure 2B in the direction opposite from which it was formed (i.e., infected → test positive), implies a change in the specific role (focal subset or reference class) of previously associated categories (or a change of *focus*; Dubois and Prade, 1997; Baratgin and Politzer, 2010), or a change in the direction of the causal link (test positive → infected).

This process can be facilitated with questions which guide reasoners through the search and selection process, for example, with integer pair question formats which prompt the reasoner for two separate numbers rather than a single percentage, and perhaps even more so if the reference class is prompted prior to the focal subset (Girotto and Gonzalez, 2001). It is interesting to note that with natural frequencies this symmetrical confusion is reduced for the first term of the integer pair (“6 out of 24”): “*Among infected, 6 test positive*” to “*Among positive, 6 are infected*,” though the more challenging asymmetrical inference still remains for the reference class. This is consistent with the suggestion that one of the biggest challenges may be getting reasoners focused on the correct reference set of positive testers (Evans et al., 2000; Girotto and Gonzalez, 2001). While this particular logical difficulty (backward reasoning or quantification of backward relations) has not been directly demonstrated in Bayesian word problems, similar explanations have been used to successfully account for performance in other reasoning tasks (Evans, 1993; Barrouillet et al., 2000; Oberauer and Wilhelm, 2000; Oberauer et al., 2005; Oberauer, 2006; Waldmann et al., 2006; Sloman and Lagnado, 2015), suggesting it may also be a key stumbling block in Bayesian reasoning. This explanation might also help to explain why reasoning can be improved with manipulations which encourage the experience of a scenario from multiple perspectives, such as “interactivity” in other Bayesian tasks (Vallée-Tourangeau et al., 2015a) and the “perspective effect” in the Monty Hall dilemma (for review see Tubau et al., 2015), which would help to facilitate the backward inference. This suggestion is also in line with results of the “short menu” natural frequencies reported in Gigerenzer and Hoffrage (1995), which directly presented both (*H*&*D*) and (*D*) in the problem, thereby eliminating all arithmetic, but which led to a negligible benefit compared with “standard menu” natural frequencies which require (*H*&*D*) + (¬*H*&*D*) to compute (*D*).

#### Summary

Taken as a whole, evidence reviewed above is consistent with the claim that normative responding is generally improved by facilitating comprehension of both presented and requested information (e.g., presenting what is needed, removing what is irrelevant, using questions which guide the reasoner) and, relatedly, minimizing the number of explicit cognitive (logical and numerical) operations required to move from problem to solution. Likewise, increasing the cognitive capacity, relevant skills, or effort of the reasoner, will generally lead to more Bayesian responses. Individuals who are more drawn toward quantitative, analytical thinking are more likely to solve these problems. This summary is consistent with a general nested-sets hypothesis, which states that any manipulation which facilitates the representation of relevant set information will generally enhance performance (Barbey and Sloman, 2007). At the same time, simply representing the relevant *qualitative* relations amongst nested sets will not get you a Bayesian response. These relations must also be accurately *quantified*, along with the correctly identified backward (posterior) inference.

More generally, understanding *why* these facilitations work as they do requires consideration of the processes in which reasoners are engaged. Ultimately, a reasoner needs to provide the requested ratio in the requested form, but arriving at this point requires the successful completion of a series of intermediate subtasks. We believe that a better understanding of successful, or failed, Bayesian problem solving can be obtained by considering: (1) How a nested-set “structure” comes to be represented by a reasoner (whether transparently presented in the problem or not); (2) What additional computational requirements are required once the structure of the problem is made “transparent” (or transparently represented by a reasoner); and (3) Who is more likely to be driven toward and successfully operate over this quantified, abstract level of reasoning. In the next section we outline one suggestion of how to conceptualize these processing requirements.

## Bayesian problem solving, from comprehension to solution

**Figure 3:**
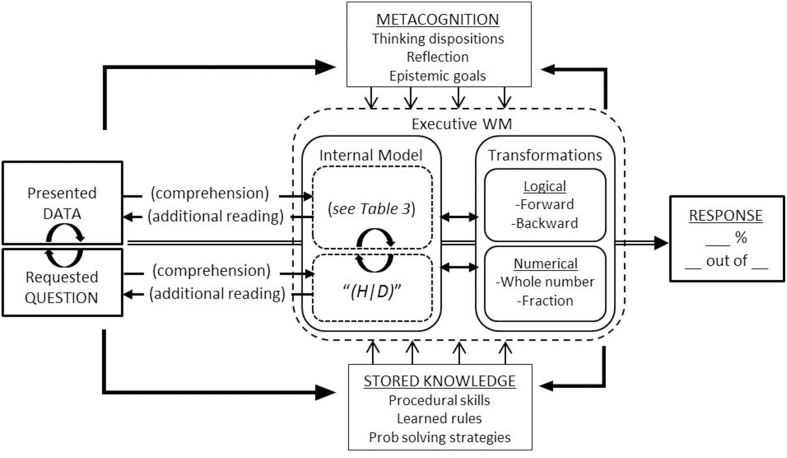
**Framework for understanding Bayesian word problem solving**. The task (left) is conceived as the presented data and the requested question. A text comprehension process gives rise to an initial internal model of the data (including inferences not directly in the text; see **Table 3**). The comprehension of the question “*(H|D)*” initiates a goal-oriented search (though internal representation and problem text) for the requested relations, along with logical and numerical computations aimed at deriving information not directly available in the text. The processing of the task also activates metacognitive dispositions as well as potentially relevant stored knowledge and skills, both of which can influence both *what* information is processed and *how* that information is processed. A continuously updated working memory also provides reciprocal feedback to metacognition and calls for additional stored knowledge if needed. The final response given will depend on a complex interaction of the task formulation, metacognition, available knowledge and skills, and the efficiency of an executive working memory. For example, metacognition can influence the effort invested into the task, while stored knowledge can influence the relative effort required for a given individual.

Solving a Bayesian word problem is a process, from the presented words and numbers, through the representations and computations invoked to transform presented information into requested ratios. As we outline below, we suggest that this process can be productively understood, at least in part, from the perspective of mathematical problem solving (for reviews see Kintsch and Greeno, 1985; Schoenfeld, 1985; LeBlanc and Weber-Russell, 1996; Mayer, 2003). On this view, the task is conceived as two interrelated processes: Text comprehension and problem solving. More specifically, successful reasoning depends essentially on comprehending the presented and requested information, and more importantly the *relation* between the two (i.e., the space between what is provided and requested). This comprehension then drives any logical or numerical computations necessary in order to reduce this space, ending with a final numerical response. A basic framework for understanding this process is outlined in Figure 3.

Two basic assumptions of this framework are (1) while the input may be the same, the (levels of) representations that reasoners operate over differ, and (2) specific individual skills or capacities and specific problem formulations can trade spaces. That is, the probability of successfully solving the problem will depend on the complexity of the provided information, as well as the number and complexity of the steps required to close this gap. Crucially, this process, and its relative difficulty, also depends on the abilities, tendencies, and skills of the problem solver (Schoenfeld, 1985, 1992; Cornoldi, 1997; Mayer, 1998, 2003; Swanson and Sachse-Lee, 2001; Passolungh and Pazzaglia, 2004). Succinctly, what is difficult for one person may not be difficult for another. In the remainder of this section, we more fully explicate this task-individual interaction as it unfolds during the reasoning process, from comprehension and computation to solution.

### Comprehension of presented and requested information

Solving a written Bayesian inference problem begins with text *comprehension*. Working memory serves as a buffer where recently read propositions and information activated in long-term memory are integrated into the internal model under construction (e.g., Just and Carpenter, 1992; Carpenter et al., 1995; Ericsson and Kintsch, 1995; Daneman and Merikle, 1996; Cain et al., 2001, 2004; Tronsky and Royer, 2003). Many inferences are automatically generated as a reader processes the symbolic words and numbers in the text (Table 3), resulting in an internal representation of the problem which may contain specific propositions included in the text itself, along with possible semantic, episodic, spatial, causal, categorical, and quantitative inferences, any of which can serve as the basis for upstream reasoning (e.g., Nesher and Teubal, 1975; van Dijk and Kintsch, 1983; Kintsch and Greeno, 1985; Murray et al., 1993; Graesser et al., 1994; LeBlanc and Weber-Russell, 1996; Vinner, 1997; Reyna et al., 2003; Reyna and Brainerd, 2008; Thompson, 2013). These levels of representations are activated to varying degrees, and may be either implicit or explicit (or not present at all) within a reasoner's model of the problem (cf. Johnson-Laird, 1983). As we return to below, given the multiple levels of information that *can* be represented, one challenge is getting a limited attention focused on the most relevant information for problem solving.

**Table 3:** **Examples of inferences and levels of encoding generated while reading a Bayesian word problem**.

		**Hypothetical knowledge and inferences**	
		**Normalized**	**Natural frequencies**
Prior knowledge or beliefs	→	Infections cause positive tests. Medical tests are usually accurate. A positive test should indicate infection. …	
Forward categorical	→	Some people are infected. Some of the infected test positive. Some of the infected do not test positive. Some of the not infected also test positive. …	
Backward categorical	→	Some of the positives are infected. Some of the positives are not infected. A positive test does not necessarily mean infected. …	
Non-integrated categorical-numerical association	→	Infected [10%] Infected-Positive [60%] Not infected [90%] Not infected-Positive [20%] …	Total [100] Infected [10] Infected-Positive [6] Not infected [90] Not infected-Positive [18] …
Forward quantitative	→	60% of 10% = 6% are both inf and pos 20% of 90% = 18% are not-inf and pos 6% + 18% = 24% of people are pos …	6 people are both inf and pos 18 people are both not-inf and pos 6 + 18 = 24 people are pos …
Backward quantitative	→	Of 24% pos, 6% are infected Of 24% pos, 18% are not infected If pos, chances of inf are 25% (6/24%) …	Of 24 positive, 6 are infected Of 24 positive, 18 are not infected If pos, chances of inf are 6 of 24 …

*Inferences may be spontaneously generated during text comprehension, or prompted as a result of the question, and may be either implicit or explicit (or not present at all) within a reasoner's model of the problem. A variety of biased responses are possible based on erroneous or irrelevant prior knowledge or beliefs, non-integrated representations, or attention to inappropriate levels of information. Inf, infected; Pos, positive test*.

It is with the reading of the question that the relevance of any initially represented problem information becomes apparent. The formulation of the question therefore plays a crucial role in Bayesian problem solving (Schwartz et al., 1991; Macchi, 1995; Girotto and Gonzalez, 2001). In general, the question provides two specific prompts: (a) verbal cues corresponding to the required categorical relations, or ratio, to be provided, and (b) the format in which the quantified response should be provided. For example, a typical natural frequency question prompts the reasoner to find two whole numbers “__ *out of* __” corresponding to “*among infected, how many positive*”; while a standard probability question demands a single percentage “__%” corresponding to “*infected if positive*”). This question comprehension triggers a goal-oriented search (through memory representations and the problem text) for the specific relations requested, along with more directed inferences and arithmetical computations targeted at deriving information not directly provided in the text (see Section Logical and Numerical Computations).

Regardless of the problem format, we expect that with relatively little effort most literate reasoners comprehend the basic situation (some people take a test for a disease), form simple categorical-numerical associations (inf[10%]; inf-pos[60%]), and make some simple forward inferences (Table 3). Ultimately, however, providing a precise Bayesian response requires accurate representation and quantification of appropriate set-subset relations (i.e., *H*&*D, D*), irrespective of the problem content. Comprehension of the *categorical* subset structure can be facilitated by presenting natural frequencies, highlighting causal structure, removing irrelevant information, providing visual diagrams, asking questions which direct attention toward relevant information, etc. Accurate comprehension of the *quantified* values (strength) of these relations is facilitated when numerical information is presented with a natural sampling structure. In the case of relative frequencies (or non-partitive probability information), on the other hand, correct *qualitative* representation of the subset structure may coincide with incorrect or incomplete *quantification* of these relations. For example, in Figure 1 it is feasible that reasoners understand the 60% hit rate to be a subset of the 10% infected, but the precise comprehension of this value requires more demanding, rule-based transformations (although some higher numerate reasoners may rather automatically perform “simple” computations such as 60 of 10% = 6%).

Individuals with more cognitive capacity will tend to more deeply processes the text (defined by the number of accurate and successfully integrated inferences; for reviews see van Dijk and Kintsch, 1983; Graesser et al., 1994), and likewise end up with a more thorough representation of the available information (both its content and structure) and the task goal as comprehended from the question. Accordingly, higher cognitive capacity or higher cognitive reflection will facilitate comprehension of a Bayesian task, at least to some extent (see Table 2). We also suggest that the processing of the task gives rise to a metacognitive assessment reflecting motivation and confidence that the problem can be solved (“*can I do this?*,” “*do I want to do this?*”), which will help to guide subsequent problem solving behavior (e.g., Schoenfeld, 1992; Cornoldi, 1997; Mayer, 1998; Thompson, 2009; also Garcia-Retamero et al., 2015).

At the same time, information from long-term memory is being integrated into working memory—including prior knowledge of causal relationships (Krynski and Tenenbaum, 2007), situational familiarity (Siegrist and Keller, 2011), or other primed categories (Kahneman et al., 1982)—which leads to different levels at which a problem can be represented (Table 3), only some of which are relevant for solving the problem. Therefore, getting focused on the relevant set relations and their numerical values, while inhibiting ultimately irrelevant contextual details and prior beliefs (e.g., about the validity of medical tests), is crucial. The ability to do so should accordingly depend in part on executive functions and working memory (see Barrett et al., 2004; Evans and Stanovich, 2013). It is further known that engaging a Bayesian problem also triggers stored knowledge associated with problem solving strategies and mathematical concepts and procedures, which act to bias attention to different levels of information within the task, for example, by leading the problem solver to analyze the text in a way which may differ from how they read stories or other news (e.g., Newell and Simon, 1972; Nesher and Teubal, 1975; Kintsch and Greeno, 1985; Anderson, 1993; Ericsson and Kintsch, 1995; Geary, 2006). In this vein, higher cognitive reflection and numeracy may also serve to bias attention toward relevant numerical information and away from irrelevant descriptive information, or more generally to relevant abstract formal relations amongst problem data rather than literal problem features (Spilich et al., 1979; Chi et al., 1981; Hegarty et al., 1995; Vinner, 1997; Peters et al., 2007; Dieckmann et al., 2009; Johnson and Tubau, 2013). This can help account for the consistent relationship between numeracy and Bayesian reasoning with natural frequencies, including interactions with non-numerical factors (Table 2).

### Logical and numerical computations

Information which is needed but not directly provided must be derived. The transformations needed to produce this information can be numerical or logical. In standard Bayesian inference tasks, numerical computations typically include whole number and/or fraction arithmetic. Whole number arithmetic is a skill that tested populations (university undergraduates; medical professionals) can be assumed to possess. At the same time, it has been shown that, even with natural frequencies, performance drops quickly when more than a single whole number addition or subtraction is required (e.g., Girotto and Gonzalez, 2001; Ayal and Beyth-Marom, 2014). Curiously, if normalized data allows the posterior relation (*H|D*) to be derived with a single whole number subtraction, performance is actually quite high, even for less numerate reasoners (Ayal and Beyth-Marom, 2014). However, it is not clear if this latter finding is due to the reduced computational demands, or from the easier representation of how to derive the standard posterior relation (e.g., by eliminating the need to perform the backward inference). While it is often assumed that fraction arithmetic (e.g., multiplying two percentages) is a skill possessed by tested populations, this may not be the case (Paulos, 1988; Butterworth, 2007; Reyna and Brainerd, 2007, 2008), as some evidence suggests (e.g., Juslin et al., 2011; Ayal and Beyth-Marom, 2014). In brief, current evidence indicates that a single whole number addition adds minimal burden to the task; more than a single operation regardless of type greatly reduces performance; and it is not clear to what extent typically tested reasoners possess the procedural skills for carrying out single multiplicative integrations.

Required computations can also be logical. As previously identified, one crucial step for solving the posterior Bayesian question which may be particularly difficult is the backward inference (*test positive*→*infection*), from the initially forward relations (*infection*→*test positive* and *no-infection*→*test positive*), or otherwise identifying the newly required reference class and focal subset (more likely prompted by the two-term integer pair question). The specific difficulties deriving and quantifying a diagnostic inference from predictive relations is well-known from causal reasoning tasks (e.g., van den Broek, 1990; Lagnado et al., 2005; Fernbach et al., 2011; Sloman and Lagnado, 2015). The asymmetry between the quantification of the relations presented and those requested requires reasoners to inhibit the precise quantifiers attached to the original relations and update the precise quantifier corresponding to the newly required relations (e.g., corresponding to the strength of the *test positive*→*infection* relation). As mentioned, natural frequencies may alleviate part of this asymmetrical confusion (for the first term of the ratio, (*H&D*); i.e., “positive among infected” = “infected among positive”), but the more challenging identification of the reference class still remains. The ability to identify and quantify this new relation should accordingly be moderated by executive functions and skill in mathematical and logical reasoning.

### Arriving at a final response

As outlined above, the final response provided by a reasoner reflects the confluence of a comprehension and problem solving process engaged by an individual with a particular set of skills and dispositions (Figure 3). The accuracy of this response will therefore depend on the level of problem comprehension and, relatedly, on the individual skills available to inspect and appropriately transform a dynamically updated internal model. A small set of rather systematic errors often account for a large proportion of erroneous responses, the most widely reported in either format being the direct selection of the hit rate, or the “inverse fallacy” (see Kahneman and Tversky, 1972; Koehler, 1996; Villejoubert and Mandel, 2002; Mandel, 2014a). Nevertheless, it is still not clear whether this results from errors understanding logical categorical relations (e.g., Wolfe, 1995; Villejoubert and Mandel, 2002) vs. superficial problem solving strategies (e.g., matching; see Evans, 1998; Stupple et al., 2013). That is, a variety of sources of failures—from erroneous comprehension of the particular relations requested to difficulties inhibiting irrelevant, previously primed information—could account for common errors, and it is not necessarily the case that the underlying cause is the same for all reasoners. The proposed framework might help to improve understanding of the causes of observed failures.

One of the main thrusts of the nested-sets hypothesis is that *if* the formulation of the problem triggers awareness of the set structural relations amongst the presented categories, *then* general cognitive resources can employ elementary set operations to mimic a Bayesian response. Musing on this possibility, Sloman et al. (2003, p. 307) suggested:

“A question that might be more pertinent is whether our manipulations changed the task that participants assigned themselves. In particular, manipulations that facilitate performance may operate by replacing a non-extensional task interpretation, like evidence strength, with an extensional one (Hertwig and Gigerenzer, 1999). Note that such a construal of the effects we have shown just reframes the questions that our studies address: under what conditions are people's judgments extensional…”

In this sense, natural frequencies (or other nested-sets facilitations) might shift reasoners into a more analytical mode of thinking due to the stronger match between presented information and the available reasoning tools of the participants, a mode we have suggested might be more automatically adopted by individuals with higher mathematical or cognitive skills. Considered from a problem solving perspective, one factor separating successful from unsuccessful reasoners may be the way they formulate and answer three crucial, interrelated questions: “*What information do I have available?*” (means), “*What information do I need to provide?*” (ends), and “*What steps do I need to take to close this gap?*” (solution plan). The relative difficulty answering these questions will of course depend on the complexity of the provided information along with the number and complexity of the required steps, and also on the individual capacities and skills of the reasoner.

More specifically, the present review suggests at least three crucial sources of difficulty for arriving at a correct Bayesian response: (1) accurately quantifying the relevant forward categorical relations of the problem, (2) accurately performing the needed backward inference, including identifying and quantifying the relevant reference class, and (3) formulating and executing and multi-step plan required for transforming presented data into the requested ratio. Each of these requirements are facilitated with natural frequencies, and become increasingly difficult with normalized data. As previously commented, performance in the latter case remains low even for participants higher in cognitive capacity or higher in numeracy, suggesting that success on these problems depends on specific skills not adequately acquired, or not spontaneously employed, by most of the participants in reviewed studies.

Hence, looked at from another direction, if the objective is to narrow the gap between human performance and Bayesian prescriptions when reasoning from explicit statistics, part of the remedy is to get participants to think more mathematically (see also Zukier and Pepitone, 1984; Schwartz et al., 1991). People are not born able to deal with abstract symbolic words and numbers. Both reading and math ability develop over time with education and practice. Even with extensive education, many individuals still fail to attain the relevant conceptual and procedural knowledge for dealing with ratios (Paulos, 1988; Brase, 2002; Ni and Zhou, 2005; Butterworth, 2007; Reyna and Brainerd, 2007, 2008; Siegler et al., 2011, 2013), a difficulty which is exacerbated when these number concepts are embedded in textual scenarios (e.g., Kirsch et al., 2002). Ultimately, therefore, deficits in explicit statistical reasoning may need to be addressed at the level of mathematics education. This remedy is not as immediate as simply reformulating a problem with natural frequencies, but in the long-term this may be a necessary way to obtain the levels of performance with which we can be satisfied.

## Future directions

The way to proceed toward a better understanding of probabilistic reasoning potentials and pitfalls depends on the specific question of interest. A variety of questions and paradigms have been addressed in this special issue on Bayesian reasoning (“Improving Bayesian Reasoning: What Works and Why?”), ranging from alternative probabilistic standards (Douven and Schupbach, 2015) to important real world issues (Navarrete et al., 2014). While many of these have focused on Bayesian word problems, other paradigms have also been discussed including “uncertain deduction” (Cruz et al., 2015; Evans et al., 2015), the Monty Hall Dilemma (Tubau et al., 2015), and the Sleeping Beauty problem (Mandel, 2014b) (for brief overviews see Mandel, 2014a; Girotto and Pighin, 2015; Juslin, 2015; McNair, 2015; Vallée-Tourangeau et al., 2015b). With respect to Bayesian word problems, many authors have expressed similar views regarding the problem-solving nature of these tasks (e.g., McNair, 2015; Sirota et al., 2015c; Vallée-Tourangeau et al., 2015b), which echo many themes presented in this review. In what follows, we offer suggestions on ways to progress in this later, problem-solving paradigm, but which may also be applicable to other paradigms as well.

Moving forward, we believe there is a need to shift perspective from the facilitators of Bayesian reasoning to more process-oriented measures aimed at uncovering the strategies evoked by successful and unsuccessful reasoners, and the stages in the problem solving process at which these differences emerge (for one proposal see De Neys and Bonnefon, 2013). To this general end, we suggest that tools from the mathematical problem solving approach might be productively applied to research on Bayesian reasoning. For example, the “moving window” (Just et al., 1982; see also De Neys and Glumicic, 2008) and online recognition paradigms (Thevenot et al., 2004; Thevenot and Oakhill, 2006) can be used to assess comprehension at different stages of problem solving, as well as *when* calculations are made throughout the reasoning process. These methods, which control or limit access to specific pieces of information, can be applied to help determine the relative difficulty of representing vs. quantifying relevant structural relations, both forward and backward.

Other process methods such as eye-tracking and recall tests are also frequently used to measure how attention is allocated during the problem solving process, which can be used to gauge the weight a reasoner assigns to different pieces of information in the text (Mayer, 1982; Hegarty et al., 1992, 1995; Verschaffel et al., 1992; for overviews see Mayer et al., 1992; LeBlanc and Weber-Russell, 1996; also De Neys, 2012). The use of protocol and error analyses have also proven effective in studies of mathematic problem solving and other areas of decision making and reasoning (e.g., Kuipers and Kassirer, 1984; Chi, 1997; Arocha et al., 2005; De Neys and Glumicic, 2008; Kingsdorf and Krawec, 2014), but apart from some notable exceptions (e.g., Gigerenzer and Hoffrage, 1995; Zhu and Gigerenzer, 2006) have thus far played only a limited role in Bayesian reasoning research. These methods can offer substantial insight into the level of information and cognitive processes that successful vs. unsuccessful reasoners engage (see also McNair, 2015). These tools can also be adapted to address questions about how participants are interpreting the tasks given to them, and the extent to which they are attempting to produce precisely computed responses vs. numerical estimates based on future uncertainties.

Finally, we suggest that these approaches be adopted alongside a strong commitment to individual differences (e.g., Stanovich et al., 2011; Del Missier et al., 2012; De Neys and Bonnefon, 2013; Klaczynski, 2014). More specifically, processing measures should look not only to establish where in the reasoning process correct and incorrect solvers depart, but also as a function of specific individual differences in ability, disposition, and requisite skills. Methods from mathematical problem solving could help to confirm or clarify existing proposals for the relevance and relative influence of these individual differences (De Neys and Bonnefon, 2013; Klaczynski, 2014).

## Conclusion

Successive waves of Bayesian reasoning research have gradually revealed that non-Bayesian responses in statistical and probabilistic word problems arise not out of biased heuristics guiding belief revision, but rather out of failed analytical processing operating over specific task structures. Even the simplest Bayesian word problems are not solved automatically, but rather involve deliberate analytical processing of the verbal and numerical structure of the task, and the subsequent logical and numerical transformations of presented data into requested relations. The formulation of the task can influence the specific types and number of inferences required to solve the problem. Hence, reducing the distance between problem and solution (mental steps) and, independently, making clear what is relevant for problem solving will generally facilitate performance. At the same time, individual differences will moderate the effect of these computational demands, as the effort required is relative to the availability of cognitive resources and relevant stored knowledge. That is, reducing processing demands and/or increasing processing resources are two complimentary means to the same end—a Bayesian response.

We have argued that a better understanding of this task-individual pair can be gained by shifting attention to the processing requirements needed to compute the Bayesian response, and the processing strategies which may be adopted by different reasoners. The proposed account, borrowed from the mathematical problem solving literature, suggests that this begins with text comprehension, an inferential and integrative process which draws on cognitive capacity and previous knowledge and skills. This gives rise to an initial problem representation, along with metacognitive assessments, which serves as the basis for the subsequent question comprehension and problem-solving behavior. We have also identified two crucial factors in this process which are likely to cause particular difficulty for many reasoners: (1) accurately *quantifying* the relevant structural relations amongst hierarchically embedded subset categories, and (2) quantifying the *backward inference* mandated by the asymmetrical direction of presented (infection → test positive) and requested (test positive → infection) information. Accordingly, interventions targeting these factors are likely to have the greatest success (i.e., natural frequencies, familiar causal relationships, guided questions), and task-relevant individual skills and abilities (numeracy, logical capacity, disposition toward analytical thinking) are likely to interact with the effectiveness of these interventions.

Given the multiple representations that Bayesian problems afford—spatially as nested sets, numerically as proportions, formally in Bayes theorem—they offer a natural link to theories of reasoning with proportional information. Accordingly, we have suggested that understanding why individuals succeed or fail on these problems can be partially anchored in the field of mathematical cognition, which has long emphasized the difficulties in learning and using ratio information, along with the importance of metacognition and executive working memory for successfully integrating different set-subset relations and for dealing with numerical information in varying contexts and formats. We believe that this complimentary perspective, and the tools it employs, can help guide a more process-oriented approach aimed at more precisely understanding where reasoning with explicit categorical and numerical information goes astray, and how the individual reasoner can be redirected to align with Bayesian norms.

### Conflict of interest statement

The authors declare that the research was conducted in the absence of any commercial or financial relationships that could be construed as a potential conflict of interest.

## Footnotes

^1^In this review we do not address the distinction between logical and subjective Bayesianism, nor do we refer to situations involving a dynamic cohesion criterion or the conditioning principle (see Baratgin and Politzer, 2006) in which other normative standards may apply (for discussion on the normative issue see Gigerenzer, 1991; Koehler, 1996; Vranas, 2000; Baratgin and Politzer, 2006, 2010; Douven and Schupbach, 2015).

^2^Information in [brackets] was not present in original text, but is included in examples in this review to ease cross-problem comparisons. [BR] = base rate, [FPR] = false-positive rate. Implicit in this example is the hit rate [HR] = 1.

^3^*Numeracy* is generally defined as the ability to work with basic numerical concepts, including the comprehension and manipulation of simple statistical and probabilistic information (for reviews see Reyna and Brainerd, 2008; Lipkus and Peters, 2009; Reyna et al., 2009; Peters, 2012). One of the most common measures used in reasoning and decision making studies is the 11-item Lipkus et al. (2001) numeracy scale, which assesses the ability to compare the relative magnitude of ratios, to convert between statistical formats, and to perform simple calculations using frequency ratios and percentages. Other measures of numeracy can be found in, for example, Cokely et al. (2012) and Peters et al. (2007).
